# Mortality and comorbidities in a Nationwide cohort of HIV-infected adults: comparison to a matched non-HIV adults’ cohort, France, 2006–18

**DOI:** 10.1093/eurpub/ckae031

**Published:** 2024-02-26

**Authors:** Alexandre Vallée, Catherine Majerholc, David Zucman, Jean-Michel Livrozet, Caroline Laurendeau, Stéphane Bouée, François Prevoteau du Clary

**Affiliations:** Department of Epidemiology and Public Health, Foch Hospital, Suresnes, France; Department of Internal Medicine, Réseau Ville-Hôpital Val de Seine, Foch Hospital, Suresnes, France; Department of Internal Medicine, Réseau Ville-Hôpital Val de Seine, Foch Hospital, Suresnes, France; Department of Infectious and Tropical Diseases, Edouard Herriot Hospital, Hospices Civils de Lyon, Lyon, France; Cemka, Bourg-la-Reine, France; Cemka, Bourg-la-Reine, France; Department of Social Medicine and Sexual Health, CHU Toulouse, Toulouse, France

## Abstract

**Background:**

Human immunodeficiency virus (HIV) remains a significant cause of morbidity and mortality worldwide. The aim of this study was to describe the mortality rate and associated comorbidities in a nationwide population-based cohort of persons living with HIV (PLWHIV) and to compare it with mortality in an age and gender-matched cohort of non-HIV individuals in France.

**Methods:**

Using data from the French national health data system, we identified and included 173 712 PLWHIV (66.5% men) and 173 712 non-HIV participants (66.5% men) matched for age and gender. PLHIV were identified based on ICD-10 HIV diagnoses, HIV-specific laboratory tests, and/or prescriptions for antiretroviral therapy specific to HIV. Hazard ratios (HRs) of mortality were assessed using multiple Cox regression models.

**Results:**

During the 13 years of follow-up (2006–18), we observed 20 018 deaths among PLWHIV compared with 6262 deaths among non-HIV participants (11.52% vs. 3.60%, *P* < 0.001). The over-mortality of PLWHIV was expressed by univariable HR = 2.135 (2.072–2.199), which remained significant after adjustment for region, Complementary Universal Health Insurance and AME, with multivariable HR = 2.182 (2.118–2.248). The results remained significant after adjusting for comorbidities, including infectious diseases [HR = 1.587 (1.538–1.638)]. Notably, PLWHIV were more importantly associated with mortality in women [HR = 2.966 (2.767–3.180)], compared in men [HR = 1.961 (1.898–2.027)].

**Conclusion:**

Although the life expectancy of PLWHIV has globally increased, the causes of death should be prioritized in prevention policies and care management. Gender-specific policies should be highlighted, as we observed a higher impact of HIV mortality in women.

## Introduction

The human immunodeficiency virus (HIV) continues to be a significant global health concern, causing a substantial burden of illness and death.^1^ Presently, there are >36 million individuals worldwide living with HIV/AIDS, and there has been a decline in overall HIV-related mortality.^1^ To combat the pandemic, the Joint United Nations Program on HIV/AIDS (UNAIDS) introduced the Fast-Track strategy, aiming to reduce HIV-related deaths to less than five hundred thousand by 2020, representing a 75% decrease from the mortality observed in 2010. Additionally, the goal is to achieve a 90% reduction in HIV-related deaths for each country between 2010 and 2030.^2^

Over the years, antiretroviral therapies (ART) have significantly improved, becoming less toxic, more effective, and demonstrating higher adherence rates.^3^ Consequently, the survival rate for patients receiving ART has increased.^3^ As a result, HIV is transforming into a manageable chronic disease, with the life expectancy of HIV-infected individuals on ART approaching that of the general population.^4^ However, despite improved access to ART, only a few countries have experienced a significant reduction in HIV mortality since 2000. Additionally, the average number of years that individuals aged 20 can expect to live remains approximately two-thirds of that in the general population.^1^^,^^4^

It is worth noting that certain cohorts of people living with HIV (PLWHIV) under proper care have demonstrated a considerable decrease in mortality, but they may not be entirely representative.^5^^,^^7^^,^^8^ Furthermore, PLWHIV experience higher mortality rates compared with the general non-HIV population, both due to AIDS-related causes and non-AIDS-related causes of death.^5–10^ PLWHIV are also more susceptible to various comorbidities, such as cardiovascular diseases, chronic viral hepatitis and cancers.^10^ These comorbidities may be linked to other associated risk factors, side effects of ART or the disease itself.

Measuring patterns of HIV/AIDS incidence, prevalence and mortality continues to be a significant challenge. This is primarily due to inadequate nationwide registration of cohorts, incomplete controlled cohorts of non-HIV populations and the complexities of disease modeling and methodological limitations.^11^ Thus, the purpose of the study was to describe the mortality rate and associated comorbidities in a nationwide population-based cohort of persons living with HIV infection and to compare with mortality in an age and gender-matched cohort of non-HIV people in France.

## Methods

### Data from the French National Insurance

The study utilized data from the French National Insurance Databases, known as the ‘Système National des Données de Santé’ (SNDS). This comprehensive database records healthcare expenditure reimbursements for all individuals covered by French health insurance, encompassing over 65 million inhabitants. The universal health insurance system covers all residents born in France, whether French or foreign, as well as legal immigrants.

The database contains individual-level information on all reimbursed medication treatments provided outside of hospitals, and it includes data on various socio-demographic factors such as date of birth, gender, and whether the person is covered by the Complementary Universal Health Insurance (CMU-c). The CMU-c offers free healthcare access to individuals with low annual income. Since not all medical expenses are fully reimbursed, individuals in France typically opt for complementary insurance to cover the remaining costs. However, those with low income receive an equivalent of this complementary insurance, the CMU-c, free of charge.

Additionally, the State Medical Aid (AME) system provides free healthcare access to foreigners in an irregular situation, subject to residency and resource conditions. The data in this database are fully linked and available from as early as 2008, offering valuable insights into healthcare consumption patterns in France.

Patient data were irreversibly anonymized using a double anonymizing algorithm. The study was assessed by the Expert Committee for Research, Studies and Evaluations in the Field of Health of the French Ministry of Higher Education, Research and Innovation.

### Study population

This study is a retrospective analysis of PLWHIV identified in the French SNDS database between 2006 and 2018, with a follow-up to 2019. PLWHIV have been matched to a non-HIV population (ratio 1:1) with the same age and gender.

COCOVIH study draws upon anonymized records from the SNDS. PLHIV were identified based on ICD-10 HIV diagnoses, HIV-specific laboratory tests and/or prescription for ART specific to HIV. Adults patients living with HIV were included between 2006 and 2018 based on an ICD-10 diagnosis of HIV mentioned as a reason for long-term care (so-called ‘ALD 7, affection longue durée n°7’) or reason for hospitalization (the codes selected were: B20, B21, B22, B23, B24, F024 and Z21), or an HIV-specific laboratory test [genotypic drug resistance test, or genotypic human leukocyte antigen (HLA) *B5701 test], or at least three prescriptions for HIV-specific ART in 1 year over the period 2006–18. Excluded were recipients of ART without corresponding HIV diagnosis, notably HIV-negative individuals on pre-exposure prophylaxis (with emtricitabine/tenofovir dual therapy) and HIV-negative patients treated for hepatitis B with tenofovir monotherapy.

**Figure 1:**
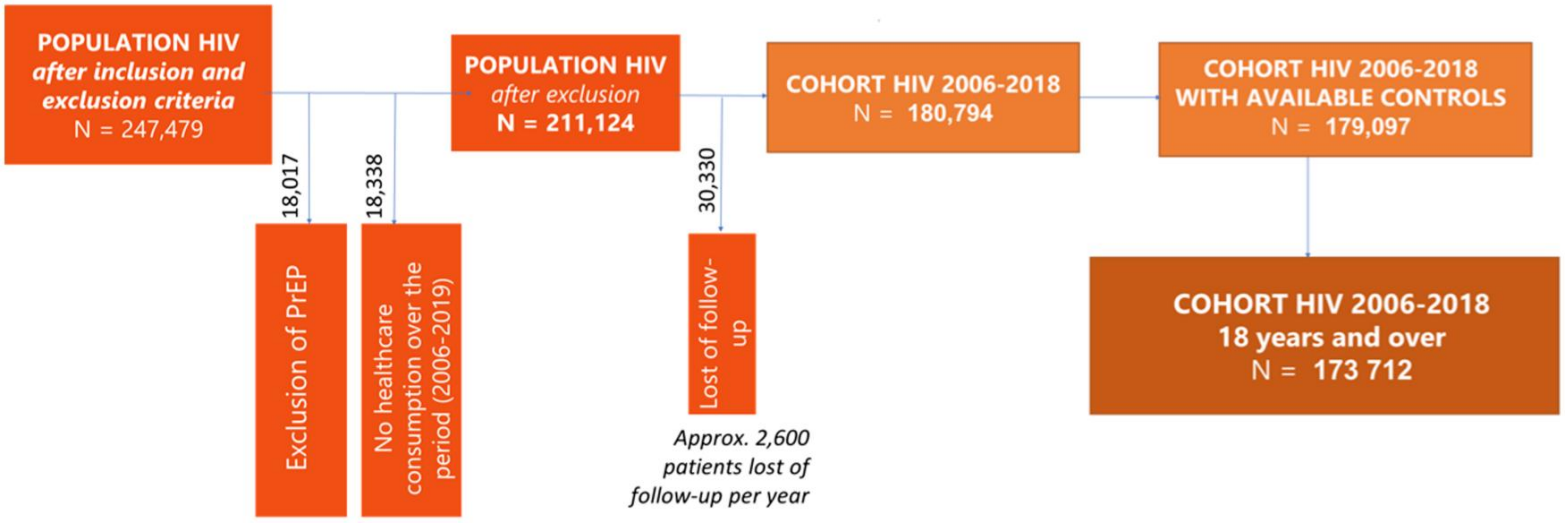
Flowchart of the population selection

An age and gender-matched control group (1:1) with no criteria for HIV infection was also included between 2006 and 2018. Each control was identified on the same date as the PLWHIV and followed up from this date (figure 1).

PLHIV were selected with an algorithm described in Supplementary file S1. The criteria to identify HIV patients using this algorithm were a full healthcare coverage for HIV, HIV-related hospitalization (ICD-10 codes: B20, B21, B22, B23, B24, F02.4, Z20.6, Z21), ART reimbursement, HIV-related biology test (i.e. genotypic resistance testing to antiretrovirals by sequencing the reverse transcriptase gene and the viral protease gene, or genotypic resistance testing to antiretroviral drugs by sequencing the envelope gene). Patients with only ART reimbursement of ATC J05AR03 were excluded from the analyses after 1 January 2016 (figure 1).^12^

### Data extraction

Data were extracted for each included participant for age, gender, date of death (if deceased) and any eligibility for ALD 7 status. Healthcare resources were extracted, such as hospitalizations, consultations, medical procedures and tests, and medications. The comorbidities were identified based on either chronic disease identified in hospital discharge summaries, ALD status or long-term medication prescription for vascular prevention or other disorders. Associated diseases were documented based on ICD-10 codes in hospital discharge summaries.

### Statistical analyses

Mean, standard deviation, median, minimum and maximum were used to describe quantitative variables, and percentages were used for qualitative variables. Bivariate analyses were conducted using χ^2^-tests to assess qualitative variables with Yates continuity correction or Fisher’s exact test for sample sizes <5. For quantitative variables, a Student’s *t*-test or analysis of variance was performed.

For the survival analyses, an index date was defined as the first date of identification of the HIV infection or 1 January 2006, for patients with full healthcare coverage for HIV before 2006. Overall survival of patients was defined as the time from the index date until death or 31 December 2018. Survival was analyzed with Kaplan–Meier curves. A multivariate Cox model analysis was performed to explore the impact of comorbidities on overall mortality. A univariate Cox model was conducted to estimate the hazard ratio (HR) of PLWHIV compared with the control group.

The first series of 11 Cox models were run with two covariates: (i) the membership of subjects to the PLWHIV group or the control groups and (ii) each of the 11 comorbidities. The HR comparing the PLWHIV group to the control groups adjusted for each of the 11 comorbidities can then be compared with the HR of the univariate analysis. The difference between these two HRs represents the attributable risk of comorbidity on the over-mortality of HIV disease.

A second series of 11 Cox models were run, and comorbidities were additionally introduced one by one in a specific order: The first comorbidity introduced was the one that led to the highest decrease in the HR comparing the PLWHIV group to the control groups. The second comorbidity led to the highest decrease in the HR while adjusting for the first comorbidity. The third comorbidity led to the highest decrease in the HR while adjusting for the first and second comorbidities. The remaining comorbidities were introduced in the same manner based on the decreased magnitude of the HR while adjusting for the previous comorbidities.

All statistical analyses were performed using SAS© software Version 9.4 (Cary, USA).

## Results

We included 173 712 PLWHIV (66.5% men) and 173 712 non-HIV participants (66.5% men) matched for age and gender (table 1). The mean age of the two populations was 41.8 (SD: 11.4) years old. PLWHIV were more frequently covered by the CMU-c additional public health insurance for low-income people and the AME additional public health insurance for illegal immigrants and mainly resided in the region of the capital of France (table 1). Repartition of PLWHIV is shown in Supplementary file S2.

**Table 1:** Description of the population

	PLWHIV	Controls
	*N*=173 712	*N* = 173 712
Age at index date		
Mean (SD)	41.8 (11.4)	41.8 (11.4)
Median/min/max	41.0/18.0/98.0	41.0/18.0/98.0
Quartile 25/quartile 75	34.0/48.0	34.0/48.0
Gender		
Men	115 603 (66.5%)	115 603 (66.5%)
Women	58 109 (33.5%)	58 109 (33.5%)
Covered by the CMU-c at the index date		
No	135 596 (78.1%)	165 968 (95.5%)
Yes	38 116 (21.9%)	7744 (4.5%)
Covered by the AME at the index date		
No	168 461 (97.0%)	172 931 (99.6%)
Yes	5251 (3.0%)	781 (0.4%)
Region of residency		
ND	1616	808
Grand-Est	8269 (4.8%)	13 334 (7.7%)
Nouvelle-Aquitaine	12 392 (7.2%)	15 663 (9.1%)
Auvergne-Rhone-Alpes	13 304 (7.7%)	19 609 (11.3%)
Bourgogne-Franche-Comte	3685 (2.1%)	6612 (3.8%)
Bretagne	5116 (3.0%)	8235 (4.8%)
Centre-Val-de-Loire	4932 (2.9%)	6518 (3.8%)
Ile de France	65 829 (38.3%)	36 270 (21.0%)
Occitanie	14 293 (8.3%)	15 811 (9.1%)
Hauts-de-France	6877 (4.0%)	13 449 (7.8%)
Normandie	4994 (2.9%)	7962 (4.6%)
Pays de la Loire	6203 (3.6%)	8912 (5.2%)
Provence-Alpes-Cote d'Azur + Corse	17 623 (10.2%)	15 182 (8.8%)
TOM	8574 (5.0%)	5335 (3.1%)
Pays Etrangers	5 (0.0%)	12 (0.0%)
Ischemic cardiopathy	15 149 (8.7%)	7073 (4.1%)
Unstable angina	3330 (1.9%)	1723 (1.0%)
Myocardial infarction	4232 (2.4%)	2277 (1.3%)
Ischemic heart disease	14 210 (8.2%)	6316 (3.6%)
Percutaneous revascularization of the coronary arteries	6465 (3.7%)	3689 (2.1%)
Stroke and transit ischemic attacks	5182 (3.0%)	2800 (1.6%)
Stroke (history of)	4226 (2.4%)	2044 (1.2%)
Transit ischemic attacks (history of)	1454 (0.8%)	1013 (0.6%)
Heart failure	6212 (3.6%)	2824 (1.6%)
Peripheral artery disease	5321 (3.1%)	2548 (1.5%)
Hypertension	46 150 (26.6%)	33 992 (19.6%)
Dyslipidemia	28 664 (16.5%)	24 221 (13.9%)
Thrombo-embolic events	3649 (2.1%)	1557 (0.9%)
pulmonary embolism	2805 (1.6%)	1130 (0.7%)
Respiratory obstructive diseases	29 155 (16.8%)	17 522 (10.1%)
Diabetes	16 460 (9.5%)	12 196 (7.0%)
Hepatitis C	22 447 (12.9%)	840 (0.5%)
Hepatitis CB	9905 (5.7%)	324 (0.2%)
Infectious diseases (history of)	66 536 (38.3%)	14 041 (8.1%)
Cancer	11 605 (6.7%)	9020 (5.2%)
Kidney diseases and/or end stage kidney disease	2339 (1.3%)	703 (0.4%)
Kidney diseases	1878 (1.1%)	638 (0.4%)
Mood disorders	47 474 (27.3%)	26 771 (15.4%)
Neurotic disorders	53 314 (30.7%)	30 457 (17.5%)
Mental and behavioral disorders due to psychoactive substance use	23 454 (13.5%)	7475 (4.3%)

PLWHIV presented a higher proportion of comorbidities compared with non-HIV participants, including cardiovascular diseases, history of stroke (2.4% vs. 1.0%, *P* < 0.001), hypertension, viral hepatitis, history of infectious disease and cancer, as well as a higher rate of diabetes (table 1).

**Figure 2:**
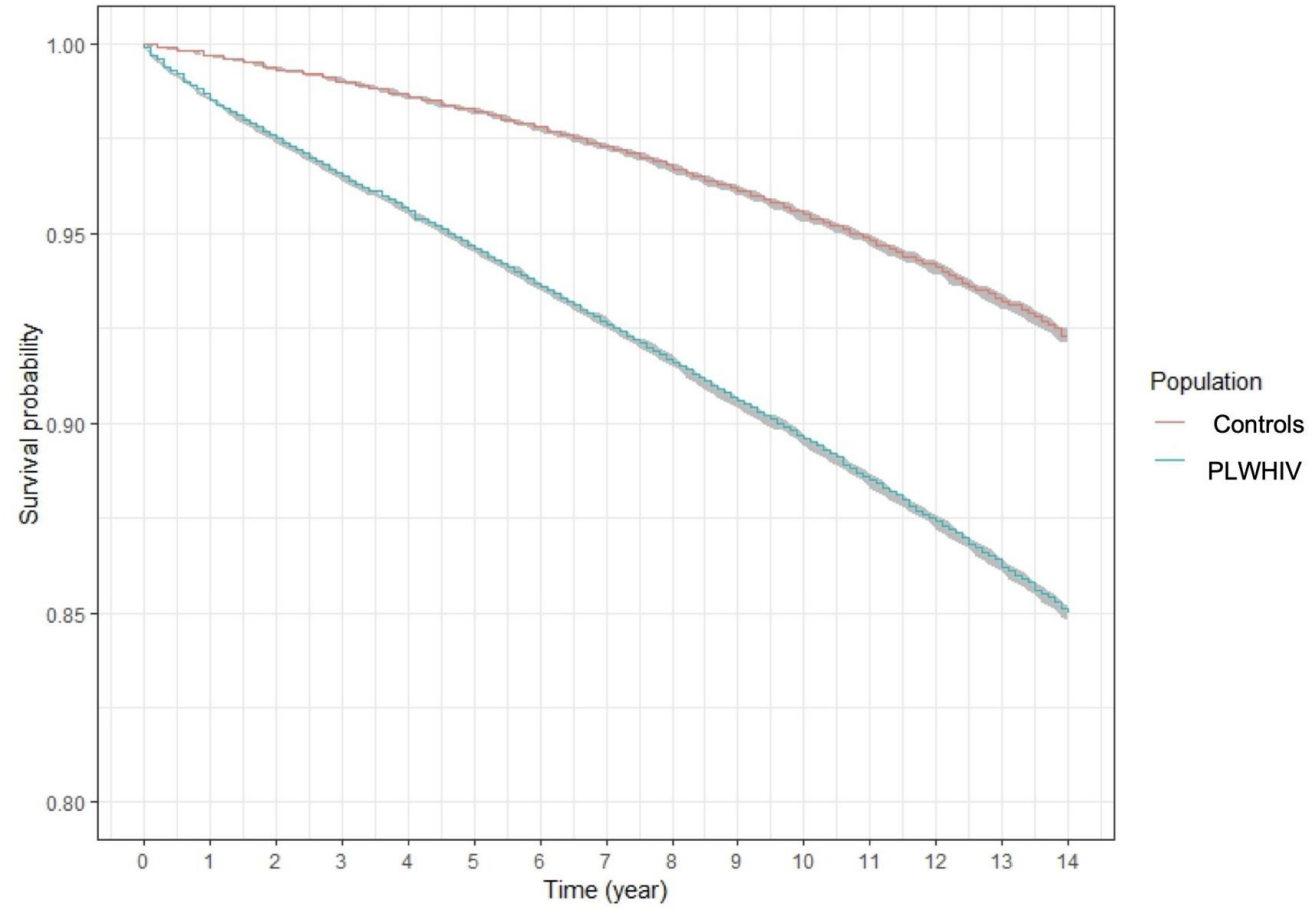
Kaplan–Meier survival curves for the PLHIV and the control group

During the 13 years of follow-up (2006–18), 20 018 PLWHIV deaths were observed compared with 6262 deaths among the non-HIV participants (11.52% vs. 3.60%, *P* < 0.001) (figure 2). At 3 years of follow-up, the survival rate of PLWHIV was 96.5% [95% confidence interval (CI) 96.4–96.5] compared with 98.9% (95% CI 98.9–99.0), at 5 years of follow-up, it was 94.5% (95% CI 94.5–94.6) compared with 98.2% (95% CI 98.1–98.3) and at 10 years of follow-up, it was 89.6% (95% CI 89.4–89.7) compared with 95.4% (95% CI 95.2–95.5) (table 2). The over-mortality of PLWHIV was expressed by univariable HR = 2.135 (2.072–2.199), which remained significant after adjustment for region, CMU-c and AME, with multivariable HR = 2.182 (2.118–2.248) (table 2).

**Table 2:** Results of the Cox model with adjustment separately on each comorbidity

	HR PLWHIV/controls	95% CI		HR decrease, %
		Inf	Sup	
Without adjustment	2.135	2.072	2.199	
Adjustment on				
Infectious diseases	1.587	1.538	1.638	48
Hepatitis C	1.791	1.736	1.847	30
Psychiatric disorders	1.950	1.893	2.009	16
Ischemic cardiopathy	2.086	2.025	2.149	4
Hepatitis B	2.063	2.002	2.126	6
Thrombo-embolic events	2.089	2.028	2.152	4
Peripheral artery disease	2.105	2.043	2.168	3
Cancer	2.106	2.045	2.17	3
Obstructive diseases	2.108	2.047	2.172	2
Stroke	2.121	2.059	2.185	1
Kidney diseases	2.134	2.072	2.199	0

After adjustment for infectious diseases, the HR decreased from 2.135 to 1.587 (1.538–1.638), *P* < 0.0001. After adjustment for hepatitis C, the HR decreased from 2.135 to 1.791 (1.736–1.847). The adjustment for other comorbidities led to smaller decreases in the HR (table 2).

The cumulative adjustments for comorbidities resulted in a significant decrease in the HR for infectious diseases [HR = 1.587 (1.538–1.638)], hepatitis C (in addition to infectious diseases) [HR = 1.393 (1.349–1.439)] and psychiatric disorders [HR = 1.354 (1.310–1.398)]. However, additional comorbidities led to much lighter decreases in the HR (1.341 for ischemic cardiopathies, 1.334 for hepatitis B), and the HR remained stable for the remaining comorbidities.

When considering stratification by gender, the over-mortality of PLWHIV was higher for women, after adjustment for region, CMU-c and AME, HR = 2.966 (2.767–3.180), than for men, HR = 1.961 (1.898–2.027) (Supplementary file S2). In both genders, infectious diseases and hepatitis C were mainly associated with mortality (Supplementary file S2). The over-mortality of PLWHIV was also higher in young people compared with older individuals (Supplementary file S2).

## Discussion

The introduction of new therapies like ART has led to a significant decrease in mortality rates among PLWHIV with high CD4 levels on HAART, aligning with rates seen in the general population.^13^^,^^14^ However, our study demonstrated that the overall mortality rate of PLWHIV remained higher than age and gender-matched non-HIV individuals (11.52% vs. 3.60%, *P* < 0.00). This outcome is consistent with previous research.^5–8^^,^^10^^,^^15–17^ For instance, a Korean study found that the mortality of PLWHIV was five to six times higher than the general population,^10^ while in Spain, the mortality rate was 7.4 (95% CI 6.0–9.0) times higher for the same age group and gender during the period of 2014–18.^8^ Another study in France showed higher mortality rates in PLWHIV across all age groups compared with the general population.^18^

The main cause of mortality among PLWHIV was AIDS-related, accounting for over 50% of overall deaths in England,^5^ Korea^10^ and Columbia.^7^ In contrast, other countries showed a decrease in HIV-related deaths, such as Spain showing 64% of the overall mortality for the period 1999–2003 and 35% for the period 2014–18,^8^ and also in Japan^19^ but depending on territories and specific populations in the USA.^16^

In Spain, excess mortality among PLWHIV was primarily associated with drug addiction and liver disease,^10^ which may be linked to a history of drug use in this population.^13^ Nevertheless, our study also found other causes of mortality among PLWHIV, such as infectious diseases, psychiatric disorders and cardiovascular diseases.

High-risk behavioral attitudes and activities, including injected drug use, were associated with mortality in PLWHIV.^20^^,^^21^ Injected drug use posed a risk for addiction, overdose and co-infections like hepatitis C and B. Additionally, it was linked to suicide, injuries, tobacco smoking and heavy alcohol consumption.^22^

PLWHIV exhibited a higher consumption of tobacco smoking, alcohol, and recreational drugs than the general population, contributing to excess mortality from infectious diseases.^23^ Psychiatric disorders were significant contributors to mortality, with suicide being a major cause of death among PLWHIV.^10^ A recent meta-analysis showed that suicide ideation prevalence was 22.4% in PLWHIV,^24^ and the risk of successful suicide 100-fold than in the general population.^25^ The stigma associated with chronic therapy like HAART might impact personal relationships and mental health.^25^

Furthermore, PLWHIV were at high risk of cardiovascular diseases and risk factors such as hypertension.^17^^,^^26^^,^^27^ HIV infection itself was identified as an independent risk factor for cardiovascular disease, with ART use potentially leading to metabolic abnormalities that increase cardiovascular risk. Several studies have shown that HIV infection is an independent risk factor for cardiovascular disease, including stroke, myocardial infarction and heart failure.^26^^,^^28^ The possible mechanism underlying this relationship could be the HIV tropism in cardiac myocytes and inflammatory responses triggered by HIV.^28^^,^^29^ ART chronic use could be associated with metabolic abnormalities leading to cardiovascular risk in PLWHIV.^30^ As explained above, PLWHIV showed a high prevalence of tobacco smoking, hypertension and dyslipidemia.^31^

Moreover, by the improvement of life expectancy of PLWHIV, the reduction of infectious diseases and changes in life behaviors, cardiovascular disease naturally increases as a cause of mortality as observed in the general population.^26^

Our study revealed that the overall mortality rate was higher among women with HIV compared with men [HR = 2.966 (2.767–3.180) vs. HR = 1.961 (1.898–2.027)]. This finding is consistent with prior research.^8^^,^^10^^,^^16^

Women exhibited a higher excess mortality in relative terms because, in the general population, women have lower mortality rates than men.^32^ This could be explained by the fact that women tend to take fewer risks and take better care of their health. However, this may not hold true for HIV-infected women, as they face higher levels of disadvantage in terms of healthcare access and quality.^33^^,^^34^ Moreover, women may experience more adverse effects related to ART, including hepatotoxicity,^35^ which could lead to mortality-related liver disease.^10^ Moreover, women also have a higher incidence of AIDS-related bacterial infections compared with men,^5^^,^^36^ with black women in the USA accounting for a significant portion of new infections among women.^37^

Mortality showed a significant association with HIV status, with an inverse age relationship. Mortality rates were higher among PLWHIV in younger age groups (18–30 years) compared with older stages. As our study matched the population for the same age groups, it indicates the importance of the impact of HIV infection on mortality at a young age. This finding could be related to the higher rates of suicide and psychiatric disorders observed among young PLWHIV.^38^

Furthermore, since 2003, the number of HIV-related deaths has declined by 64% in overall age groups and by 74% among children aged 0–9 years, but the decline was only 10% among adolescents.^39^ Several determinants could explain this observation, including restrictive laws on the age of consent for self-care, poor adherence to ART, and behavioral risks related to age, social, and economic status, which may limit adolescents' access to information and healthcare services.^40^

### Limitations

The main strengths of this study are the utilization of the SNDS database, which covers the entire HIV French population, and the comparison of this population with a matched controlled non-HIV population of the same age and gender. In France, causes of death were recorded through INSEE certificates and supported by multiple administrative sources, ensuring the accuracy and validity of death-related information. The long follow-up period spanning over 13 years allowed for the assessment of long-term survival. However, this study also has some limitations. We may have underestimated the prevalence of hypertension, diabetes and dyslipidemia because these conditions were identified only through hospital records for hospitalization and prescriptions. As a result, cases of these conditions treated outside the hospital or not requiring ART prescriptions may have been missed in our analysis.

## Conclusion

Although screening HIV tests, ART and general medications are free and reimbursed in France, the mortality among PLWHIV is higher than in a matched non-HIV population of the same age and gender. Thus, health policies should prioritize early HIV diagnosis, treatment and healthcare. Even though the life expectancy of PLWHIV has globally increased, prevention policies and care management should focus on addressing the specific causes of death. Gender-specific policies should be highlighted, as we observed a higher impact of HIV mortality in women. Special attention should be given to the treatment of the younger population to prevent early deaths. Moreover, efforts should be directed toward preventing avoidable infectious diseases through systematic and widespread vaccination, along with early treatment following guidelines. Mental health and cardiovascular prevention should be particularly investigated to prevent and reduce mortality among people living with HIV.

## Supplementary Material

ckae031_Supplementary_Data

## Data Availability

The data that support the findings of this study are available on request from the corresponding author. The data are not publicly available due to privacy or ethical restrictions. *Institutional Review Board Statement*: Not applicable *Informed Consent Statement*: A non-opposed consent was obtained for all participants. Patient data were irreversibly anonymized using a double anonymizing algorithm. The study was assessed by the Expert Committee for Research, Studies and Evaluations in the Field of Health (CEREES) of the French Ministry of higher Education, Research and Innovation. Key points Mortality among PLWHIV is higher than in a matched non-HIV population. Health policies should prioritize early HIV diagnosis, treatment, and healthcare. Special attention should be given to the treatment of the younger population.

## References

[1] GBD 2017 HIV Collaborators. Global, regional, and national incidence, prevalence, and mortality of HIV, 1980-2017, and forecasts to 2030, for 195 countries and territories: a systematic analysis for the Global Burden of Diseases, Injuries, and Risk Factors Study 2017. Lancet HIV 2019;6:e831–59. 31439534 10.1016/S2352-3018(19)30196-1 PMC6934077

[2] Ghys PD , Williams BG, Over M, et al Epidemiological metrics and benchmarks for a transition in the HIV epidemic. PLoS Med 2018;15:e1002678. 30359372 10.1371/journal.pmed.1002678 PMC6201869

[3] Lima VD , Hogg RS, Harrigan PR, et al Continued improvement in survival among HIV-infected individuals with newer forms of highly active antiretroviral therapy. AIDS 2007;21:685–92. 17413689 10.1097/QAD.0b013e32802ef30c

[4] Antiretroviral Therapy Cohort Collaboration. Life expectancy of individuals on combination antiretroviral therapy in high-income countries: a collaborative analysis of 14 cohort studies. Lancet Lond Engl 2008;372:293–9. 10.1016/S0140-6736(08)61113-7 PMC3130543 18657708

[5] Croxford S , Kitching A, Desai S, et al Mortality and causes of death in people diagnosed with HIV in the era of highly active antiretroviral therapy compared with the general population: an analysis of a national observational cohort. Lancet Public Health 2017;2:e35–46. 29249478 10.1016/S2468-2667(16)30020-2

[6] Simmons RD , Ciancio BC, Kall MM, et al Ten-year mortality trends among persons diagnosed with HIV infection in England and Wales in the era of antiretroviral therapy: AIDS remains a silent killer. HIV Med 2013;14:596–604. 23672663 10.1111/hiv.12045

[7] Eyawo O , Franco-Villalobos C, Hull MW, Comparative Outcomes and Service Utilization Trends (COAST) Study, et al Changes in mortality rates and causes of death in a population-based cohort of persons living with and without HIV from 1996 to 2012. BMC Infect Dis 2017;17:174. 28241797 10.1186/s12879-017-2254-7 PMC5329918

[8] Fontela C , Aguinaga A, Moreno-Iribas C, et al Trends and causes of mortality in a population-based cohort of HIV-infected adults in Spain: comparison with the general population. Sci Rep 2020;10:8922. 32488053 10.1038/s41598-020-65841-0 PMC7265289

[9] Marcus JL , Leyden WA, Alexeeff SE, et al Comparison of overall and comorbidity-free life expectancy between insured adults with and without HIV infection, 2000-2016. JAMA Netw Open 2020;3:e207954. 32539152 10.1001/jamanetworkopen.2020.7954 PMC7296391

[10] Park B , Choi Y, Kim JH, et al Mortality and causes of death among individuals diagnosed with human immunodeficiency virus in Korea, 2004-2018: an analysis of a nationwide population-based claims database. Int J Environ Res Public Health 2022;19:11788. 36142061 10.3390/ijerph191811788 PMC9517230

[11] Murray CJL , Ortblad KF, Guinovart C, et al Global, regional, and national incidence and mortality for HIV, tuberculosis, and malaria during 1990-2013: a systematic analysis for the Global Burden of Disease Study 2013. Lancet 2014;384:1005–70. 25059949 10.1016/S0140-6736(14)60844-8 PMC4202387

[12] Billioti de Gage S , Desplas D, Dray-Spira R. Roll-out of HIV pre-exposure prophylaxis use in France: a nationwide observational study from 2016 to 2021. Lancet Reg Health Eur 2022;22:100486. 35990255 10.1016/j.lanepe.2022.100486 PMC9386455

[13] Lewden C , Bouteloup V, De Wit S, Collaboration of Observational HIV Epidemiological Research Europe (COHERE) in EuroCoord, et al All-cause mortality in treated HIV-infected adults with CD4 ≥500/mm3 compared with the general population: evidence from a large European observational cohort collaboration. Int J Epidemiol 2012;41:433–45. 22493325 10.1093/ije/dyr164

[14] Wada N , Jacobson LP, Cohen M, et al Cause-specific mortality among HIV-infected individuals, by CD4(+) cell count at HAART initiation, compared with HIV-uninfected individuals. AIDS 2014;28:257–65. 24105030 10.1097/QAD.0000000000000078 PMC4164055

[15] de Coninck Z , Hussain-Alkhateeb L, Bratt G, et al Non-AIDS mortality is higher among successfully treated people living with HIV compared with matched HIV-negative control persons: a 15-year follow-up cohort study in Sweden. AIDS Patient Care STDS 2018;32:297–305. 30067408 10.1089/apc.2018.0015 PMC6088250

[16] Bosh KA , Johnson AS, Hernandez AL, et al Vital signs: deaths among persons with diagnosed HIV infection, United States, 2010-2018. MMWR Morb Mortal Wkly Rep 2020;69:1717–24. 33211683 10.15585/mmwr.mm6946a1 PMC7676640

[17] Yu X , Westra JR, Giordano TP, et al Assessing comorbidities and survival in HIV-infected and uninfected matched Medicare enrollees. AIDS 2021;35:1667–75. 34049353 10.1097/QAD.0000000000002963 PMC8286326

[18] Prodel M , Finkielsztejn L, Roustand L, et al Costs and mortality associated with HIV: a machine learning analysis of the French national health insurance database. J Public Health Res 2021;11:2601. 34850620 10.4081/jphr.2021.2601 PMC8958442

[19] Nishijima T , Inaba Y, Kawasaki Y, et al Mortality and causes of death in people living with HIV in the era of combination antiretroviral therapy compared with the general population in Japan. AIDS 2020;34:913–21. 32039993 10.1097/QAD.0000000000002498 PMC7170431

[20] Lai C-C , Hsu C-K, Yen M-Y, et al Monkeypox: an emerging global threat during the COVID-19 pandemic. J Microbiol Immunol Infect 2022;55:787–94. 35970757 10.1016/j.jmii.2022.07.004 PMC9352646

[21] Bragazzi NL , Kong JD, Mahroum N, et al Epidemiological trends and clinical features of the ongoing monkeypox epidemic: a preliminary pooled data analysis and literature review. J Med Virol 2022;95:e27931. 35692117 10.1002/jmv.27931

[22] Trickey A , May MT, Vehreschild J, et al; Antiretroviral Therapy Cohort Collaboration (ART-CC). Cause-specific mortality in HIV-positive patients who survived ten years after starting antiretroviral therapy. PloS One 2016;11:e0160460. 27525413 10.1371/journal.pone.0160460 PMC4985160

[23] Xu J-F , Wang P-C, Cheng F. Health related behaviors among HIV-infected people who are successfully linked to care: an institutional-based cross-sectional study. Infect Dis Poverty 2020;9:28. 32169118 10.1186/s40249-020-00642-1 PMC7068930

[24] Pei J-H , Pei Y-X, Ma T, et al Prevalence of suicidal ideation, suicide attempt, and suicide plan among HIV/AIDS: A systematic review and meta-analysis. J Affect Disord 2021;292:295–304. 34134028 10.1016/j.jad.2021.05.064

[25] Pelton M , Ciarletta M, Wisnousky H, et al Rates and risk factors for suicidal ideation, suicide attempts and suicide deaths in persons with HIV: a systematic review and meta-analysis. Gen Psychiatr 2021;34:e100247. 33912798 10.1136/gpsych-2020-100247 PMC8042999

[26] Park DY , An S, Romero ME, et al National trend of heart failure and other cardiovascular diseases in people living with human immunodeficiency virus. World J Cardiol 2022;14:427–37. 36161061 10.4330/wjc.v14.i7.427 PMC9350607

[27] Ogarkova D , Antonova A, Kuznetsova A, et al Current trends of HIV infection in the Russian Federation. Viruses 2023;15:2156. 38005834 10.3390/v15112156 PMC10674383

[28] Delabays B , Cavassini M, Damas J, et al Cardiovascular risk assessment in people living with HIV compared to the general population. Eur J Prev Cardiol 2022;29:689–99. 34893801 10.1093/eurjpc/zwab201

[29] Prendergast BD. HIV and cardiovascular medicine. Heart 2003;89:793–800. 12807864 10.1136/heart.89.7.793 PMC1767745

[30] Blanco F , San Román J, Vispo E, et al Management of metabolic complications and cardiovascular risk in HIV-infected patients. AIDS Rev 2010;12:231–41. 21179187

[31] So-Armah K , Freiberg MS. HIV and cardiovascular disease: update on clinical events, special populations, and novel biomarkers. Curr HIV/AIDS Rep 2018;15:233–44. 29752699 10.1007/s11904-018-0400-5 PMC6230511

[32] Zhao Y , Wei L, Dou Z, et al Changing mortality and patterns of death causes in HIV infected patients–China, 2013–2022. China CDC Wkly 2023;5:1073–8. 38058989 10.46234/ccdcw2023.201 PMC10696223

[33] Kowalska JD , Aebi-Popp K, Loutfy M, et al; Women Against Viruses in Europe (WAVE) Working Group. Promoting high standards of care for women living with HIV: position statement from the Women Against Viruses in Europe Working Group. HIV Med 2018;19:167–73. 29159861 10.1111/hiv.12565

[34] Burchell AN , Raboud J, Donelle J, et al Cause-specific mortality among HIV-infected people in Ontario, 1995-2014: a population-based retrospective cohort study. CMAJ Open 2019;7:E1–E7. 10.9778/cmajo.20180159 PMC6350837 30622108

[35] Clark R. Sex differences in antiretroviral therapy-associated intolerance and adverse events. Drug Saf 2005;28:1075–83. 16329711 10.2165/00002018-200528120-00003

[36] Søgaard OS , Reekie J, Ristola M, et al Severe bacterial non-AIDS infections in HIV-positive persons: incidence rates and risk factors. J Infect 2013;66:439–46. 23353671 10.1016/j.jinf.2012.12.012

[37] Ojikutu BO , Mayer K. HIV prevention among Black women in the US-time for multimodal integrated strategies. JAMA Netw Open 2021;4:e215356. 33835180 10.1001/jamanetworkopen.2021.5356

[38] Tsai Y-T , Padmalatha S, Ku H-C, et al Suicidality among people living with HIV from 2010 to 2021: a systematic review and a meta-regression. Psychosom Med 2022;84:924–39. 36162070 10.1097/PSY.0000000000001127 PMC9553271

[39] UNICEF. *Global and Regional Trends*. 2021.Available at: https://data.unicef.org/topic/hivaids/global‐regional‐trends/ (November 2023, date last accessed).

[40] Idele P , Gillespie A, Porth T, et al Epidemiology of HIV and AIDS among adolescents: current status, inequities, and data gaps. J Acquir Immune Defic Syndr 1999 2014;66(Suppl 2):S144–153. 10.1097/QAI.0000000000000176 24918590

