# Supplementary material for: Mortality and comorbidities in a Nationwide cohort of HIV-infected adults: comparison to a matched non-HIV adults’ cohort, France, 2006–18
Source: Eur J Public Health. 2024 Feb 26;34(5):879–84. doi: 10.1093/eurpub/ckae031 (PMC11430913; doi:10.1093/eurpub/ckae031)
Supplement: ckae031_Supplementary_Data [file ckae031_supplementary_data.zip › ckae031_Supplementary_Data/ejph-2023-11-om-0664-File004.docx]

**SUPPLEMENTAL FILE**

# Algorithms used for comorbidities identifications

## Infectious diseases

Infectious diseases leading to hospital stays were identified through hospital stays with ICD-10 codes for infectious diseases (A00 to B99) as primary or secondary diagnoses except:

- B20 to B24 (Human immunodeficiency virus [HIV] disease);
- B18 (Chronic viral hepatitis).

## Hepatitis B

Hepatitis B was identified through:

- A long term disease for hepatitis B (ICD-10 codes B180 and B181);
- Past hospital stays with ICD-10 codes for hepatitis B ICD-10 codes B180 and B181) as primary or secondary diagnoses:

## Hepatitis C

Hepatitis C was identified through:

- A long term disease for hepatitis C (ICD-10 code B18.2);
- Past hospital stays with ICD-10 codes for hepatitis C (B18.2) as primary or secondary diagnoses;
- A treatment specific of hepatitis C

Table 5: Treatment specific of hepatitis C

| **Name** | **ATC code** |
| --- | --- |
| telaprevir | J05AE11 |
| ribavirine | J05AB04 |
| bocéprévir | J05AE12 |
| siméprévir | J05AE14 |
| daclatasvir | J05AX14 |
| sofosbuvir | J05AX15 |
| dasabuvir | J05AX16 |
| lédipasvir, sofosbuvir | J05AX65 |
| ombitasvir, paritaprévir, ritonavir | J05AX67 |

## Ischemic cardiopathy

Ischemic cardiopathy was identified through:

- A long term disease for ischemic heart disease (ICD-10 code I20* to I25*);
- Past hospital stays with ICD-10 codes myocardial infarction (I21*) as primary or secondary diagnoses:

## Thrombo-embolic events

Thrombo-embolic events were identified through:

- Past hospital stays with ICD-10 codes pulmonary embolism (I26*) as primary or secondary diagnoses:
- Past hospital stays with ICD-10 codes thrombophlebitis (I80*) as primary or secondary diagnoses:

## Stroke and transit ischemic attacks

Stroke and transit ischemic attacks were identified through:

- A long term disease for a sequelae of stroke (ICD-10 code I69*);
- Past hospital stays with ICD-10 codes for cerebral infarction or stroke or transit ischemic attack (G45*) as primary or secondary diagnoses.

## Peripheral artery disease

Peripheral artery disease was identified through:

- A long term disease for peripheral artery diseases (ICD-10 code I70*);
- Past hospital stays with ICD-10 codes for peripheral artery diseases (I70*) as primary or secondary diagnoses:

## Cancer

Cancers were identified through a long term disease for cancer (ICD-10 code I70*).

## Respiratory obstructive diseases

Respiratory obstructive diseases was identified through:

- A long term disease for respiratory obstructive diseases (ICD-10 codes J41, J42, J43, J44, J45, J46, J47*);
- Past hospital stays with ICD-10 codes for peripheral artery diseases (J41, J42, J43, J44, J45, J46, J47*) as primary or secondary diagnoses;
- Deliveries of drugs indicated for obstructive airway diseases (ATC codes R03*)

##### Kidney diseases

Kidney diseases were identified through a long term disease for a kidney disease diseases (ICD-10 code N00* to N19*).

##### Kidney diseases and/or end stage kidney disease

Kidney diseases were identified through

- A long term disease for a kidney disease diseases (ICD-10 code N00* to N19*);
- A hospital stay for renal transplant;
- Chronic dialysis (at least 45 hospital stays for dialysis).

## Psychiatric disorders

Three psychiatric disorders are included in this category:

- Mood disorders;
- Neurotic, stress-related and somatoform disorders;
- Mental and behavioural disorders due to psychoactive substance use.

### Mood disorders

Mood disorders were identified through:

- A long term disease for mood [affective] disorders (ICD-10 codes F30* to F39*);
- Past hospital stays with ICD-10 codes for mood [affective] disorders (F30* to F39*) as primary or secondary diagnoses;
- Deliveries of antidepressants (ATC codes N06A*) or lithium (ATC codes N05AN01).

### Neurotic disorders

Neurotic disorders were identified through:

- A long term disease for neurotic disorders (ICD-10 codes F40* à F48*);
- Past hospital stays with ICD-10 codes for neurotic disorders (ICD-10 codes F40* à F48*) as primary or secondary diagnoses;
- Deliveries of anxiolytics (ATC codes N05B*).

### Mental and behavioural disorders due to psychoactive substance use

Patients suffering from mental and behavioural disorders due to psychoactive substance use were identified through:

- A long term disease for mental and behavioural disorders due to psychoactive substance (ICD-10 codes F10*, F11*, F12*, F13*, F14*, F15*, F16*, F18* and F19*);
- Past hospital stays with ICD-10 codes for mental and behavioural disorders due to psychoactive substance (ICD-10 codes F10*, F11*, F12*, F13*, F14*, F15*, F16*, F18* and F19*) as primary or secondary diagnoses;

##### Heart failure

Patients suffering from chronic heart failure were identified through:

- A long term disease for chronic heart failure (ICD-10 code I50*);
- Past hospital stays with ICD-10 codes for chronic heart failure (ICD-10 codes 50*) as primary or secondary diagnosis.

##### Hypertension

Hypertension was defined by the following variables:

- A long term disease for Hypertension (I10*, I11*, I12*, I13*, I15*);
- Past hospital stays with ICD-10 codes for Hypertension as primary or secondary diagnosis;

##### Hypercholesterolemia

Dyslipidemia was defined by the reimbursement over a period corresponding to at least 3 reimbursements on 3 different dates or at least 2 reimbursements on 2 different dates of forms suited to treatment for a 3-month period (large pack sizes) (ATC class C10*), before the index date.

##### Diabetes

Diabetes was defined by either:

- A long term disease for diabetes: E10 (Insulin-dependent diabetes mellitus) or E11 (Non-insulin-dependent diabetes mellitus);
- Past hospital stays with ICD-10 codes for diabetes as primary or secondary diagnosis.
- The reimbursement over a period corresponding to at least 3 reimbursements on 3 different dates or at least 2 reimbursements on 2 different dates of forms suited to treatment for a 3-month period (large pack sizes) (ATC class A10*), before the index date.
