# Supplementary material for: Mortality and comorbidities in a Nationwide cohort of HIV-infected adults: comparison to a matched non-HIV adults’ cohort, France, 2006–18
Source: Eur J Public Health. 2024 Feb 26;34(5):879–84. doi: 10.1093/eurpub/ckae031 (PMC11430913; doi:10.1093/eurpub/ckae031)
Supplement: ckae031_Supplementary_Data [file ckae031_supplementary_data.zip › ckae031_Supplementary_Data/ejph-2023-11-om-0664-File005.docx]

Supplementary file 2

Supplemental Figure 1: People living with HIV in France according to regions (presentation in percentage).

**Supplemental figure 2:** Results of the cox model with adjustment additively on each comorbidity stratified on gender and age.
